# “I sort of never felt like I should be worried about it or that I could be worried about it’” an interpretative phenomenological analysis of perceived barriers to disclosure by young people with coeliac disease

**DOI:** 10.1111/bjhp.12599

**Published:** 2022-05-16

**Authors:** Miranda Wheeler, Annabel L. David, Juliet Kennedy, Matthew Knight

**Affiliations:** ^1^ The Oxford Institute of Clinical Psychology Training and Research The Oxford Centre for Psychological Health, Oxford Health NHS Foundation Trust University of Oxford Oxford UK; ^2^ Children’s Psychological Medicine Oxford Children’s Hospital Oxford University Hospitals NHS Foundation Trust Oxford UK

**Keywords:** coeliac disease, disclosure, interpretative phenomenological analysis, paediatrics, psychological difficulties, stigma

## Abstract

**Objectives:**

There has been little research in the United Kingdom regarding young people's experiences of disclosure of psychological difficulties relating to coeliac disease (CD) to others, particularly healthcare professionals. This study sought to address this systematically with a focus on the lived experiences of young people with CD. This study aimed to gain insight into how paediatric gastroenterology services could improve the patient experience for those with CD and support the identification of patients who may benefit from further psychological support.

**Design:**

This study used interpretive phenomenological analysis (IPA) of patient accounts.

**Methods:**

Seven young people with CD (aged 11–16 years) were recruited from a UK hospital paediatric gastroenterology service. Semi‐structured interviews were carried out and verbatim transcripts were analysed using IPA to explore young people's experiences of CD and why they might feel able or unable to disclose psychological difficulties associated with their condition to clinicians.

**Results:**

Three superordinate themes were interpreted from the data. The first encapsulated experiences of adjusting to the diagnosis within a developmental context, including the role of adults in information provision and the importance of peer support. The second outlined experiences of managing perceived or actual stigma regarding others’ perceptions of the condition and themselves. The third incorporated perceived barriers to disclosure relating to power, safety, and beliefs about the role of medical professionals.

**Conclusions:**

Findings highlight the importance of clinicians continually providing developmentally appropriate information to young people and actively breaking down barriers to disclosure through body language and the use of clear questions regarding emotional experiences.

**Statement of contribution**

***What is already known on this subject?***

- Receiving a diagnosis of coeliac disease as an adolescent poses particular developmental challenges when adapting to a new diet and lifestyle.
- Difficulties adapting to this diagnosis can increase the risk of non‐adherence to the gluten‐free diet (GFD) and the likelihood of experiencing physical and psychological difficulties.
- Identification of young people with long‐term conditions who may benefit from psychological support is challenging and it is therefore important to understand the barriers to disclosure of psychological difficulties to healthcare professionals.

***What does this study add?***

- Young people rely on adults to scaffold their understanding of CD and how to disclose it to others.
- Reduced understanding creates difficulties cultivating support and increases experiences of stigma.
- Clinician recommendations to improve patient experience and to support disclosure are made.

## INTRODUCTION

Coeliac disease (CD) is a genetic lifelong autoimmune condition whereby ingesting gluten leads to a range of idiosyncratic symptoms including diarrhoea, abdominal pain, and chronic fatigue (Barratt et al., 2013; Green & Jabri, 2003, 2006). Treatment for CD requires the individual to maintain a strict gluten‐free diet (GFD), whereby they must exclude foods containing wheat, barley, and rye (NICE, 2014). Long‐term non‐adherence to the GFD is problematic and can result in complications such as osteoporosis, lymphoma, and small bowel cancer (Green & Jabri, 2003; Rubio‐Tapia & Murray, 2010).

Research indicates that being diagnosed with CD as a young person, of secondary school age, poses particular challenges when adapting to a new diet and lifestyle (NICE, 2014). Developmentally, this age range involves increasing autonomy from parents, growing responsibility for one's own well‐being and food‐related activities, and increasing peer pressure to conform (Ludvigsson et al., 2016). With this increased autonomy comes increased responsibility for their health and a transition away from family‐focused CD management to individual CD management (Rosen, 1995). For this transition to be successful, young people are required to develop sufficient knowledge of CD and the GFD, the consequences of non‐adherence to the GFD, and confidence in their ability to inform others about the condition and seek support when needed (Cooley et al., 2011). Lack of opportunity to develop these skills, alongside difficulties adapting to a diagnosis of CD at this developmental stage can lead to non‐adherence, poor health‐related quality of life, and psychological difficulties (Mayer et al., 1991; Mazzone et al., 2011; Schroeder & Mowen, 2014). A recent systematic review suggests that there is an increased prevalence of anxiety, depression, and somatic complaints in young people with CD, emphasizing the importance of identifying young people who may benefit from psychological support (Coburn et al., 2019).

Identification poses a challenge to services, as young people can experience difficulties disclosing psychological difficulties, exacerbated by the process of adjusting to a chronic condition (Rickwood et al., 2007; Rutter, 1995). For instance, research exploring disclosure and support‐seeking in young people with heart disease and inflammatory bowel disease (IBD) suggests that only 60% and 62.5% of individuals shared concerns related to their well‐being during routine consultations, respectively (Ashton et al., 2016; Vatne et al., 2012). Moreover, Vatne et al. (2012) found that young people were more likely to express subtle negative emotional cues during routine consultations, as opposed to clear verbalizations of distress. A systematic review by Gulliver et al. (2010) suggests that the characteristics of the treating clinician, including credibility, unhelpful responses, and being perceived as “too busy” pose possible perceived barriers to help‐seeking in 11–25‐year‐olds.

Identification of young people who are experiencing clinically significant psychological symptoms related to their CD may be further compromised by a lack of integration of psychological support and consultation within multidisciplinary teams (MDT's; Coburn et al., 2020; Crocker et al., 2020). In line with this, Coeliac UK (2018) have identified “finding the best post‐diagnosis support” as a “top 10 research priority” following consultation with people with CD, their families, and healthcare professionals. A US study reported that 49% of young people attending a paediatric CD clinic displayed clinically significant psychological symptoms, including anxiety, depression, behaviour problems, attention and concentration difficulties, and/or disordered eating or feeding problems (Coburn et al., 2020). Moreover, 68.1% of all families identified mental health consultation as one of their top two priorities when attending medical appointments (Coburn et al., 2020). However, research in a sample of 800 adults with CD across the United Kingdom suggests that emotional well‐being was rarely assessed (17.6% cases) by clinicians during follow‐up appointments (Crocker et al., 2020). This raises the question of whether young people with CD perceive that they have had the opportunity to disclose distress associated with CD during routine consultations, or whether they too perceived barriers to disclosure (Gulliver et al., 2010).

We aimed to address these issues systematically by providing a theoretically integrative account of young people's experiences of disclosing or not disclosing psychological difficulties to clinicians. Research objectives were as follows:

1. To understand what it is like for young people to have CD.
2. To explore young people's experiences of disclosing and not disclosing psychological difficulties associated with CD to clinicians in a UK paediatric gastroenterology service.
3. To explore young people's perspectives regarding any specific changes that could be made to services to promote disclosure and improve access to psychological services, if it is felt that this would be helpful for this population.

Subsequently, this study aimed to make recommendations for services based on findings, as necessary.

## METHODS

### Design

We employed an interpretive phenomenological analysis (IPA) methodology in line with our focus on lived experience, with special consideration of the interpersonal and socio‐developmental context (Smith et al., 2009).

### Research team

The lead researcher (MW, trainee clinical psychologist at the time), co‐designed the study, conducted the interviews, and took the lead role in the analysis. Three clinical psychologists co‐designed and supervised the project (AD, who has clinical and research experience in this area, JK, who has clinical experience in paediatrics, and MK, who is experienced with IPA). Please see the supporting information for further details on the reflexivity and positioning of researchers.

### Participants

We recruited participants from a tertiary paediatric gastroenterology service using purposive sampling. The service supports young people from birth to 18 years of age. However, to limit sample heterogeneity, participants were eligible to take part if they were 11–16 years old and attending secondary school; had a medical diagnosis of CD; had been under the care of the service for at least 3 months; and had attended at least one follow‐up appointment. We excluded participants if they had a co‐morbid diagnosed gastrointestinal condition and/or if they were currently receiving psychological support from any psychological service.

We screened young people attending CD consultations in the service for eligibility and, if eligible, we sent their parent/guardian a letter by post inviting the young person to participate in an interview. We sent the invitation to eligible 16‐year‐olds directly, followed by a further invitation letter 3 weeks later if they had not responded. We initially commenced recruitment in March 2020 but subsequently paused in mid‐March 2020 due to the pandemic. We resumed recruitment from September 2020 to October 2020, achieving 25% uptake (28 invited). Interested young people or their parents contacted the primary researcher and were subsequently screened and sent an information sheet. All participants were required to give consent to take part, and parental consent was required for those under the age of 16. All participants completed a demographic questionnaire.

The sample consisted of seven white British participants, five females and two males, aged 11–16 (*M* = 14 years, 2 months; *SD* = 1 year, 8 months). All participants had a medical diagnosis of CD, whereby the mean age of diagnosis was 7 years, 5 months (*SD* = 4 years, 6 months; range 1 year, 6 months–13 years, 5 months). All participants had at least one family member with CD. One participant had co‐morbid attention‐deficit/hyperactivity disorder and had previously received psychological input from the service. The remaining participants had no co‐morbid diagnoses and were not under the care of other services for their CD. Additional sample characteristics are provided in Table 1.

**TABLE 1:** Sample characteristics

	Pseudonym	Current age (y, m)	Gender	Age when diagnosed (y, m)	Family members with CD
1	Sarah	15 y, 4 m	F	10 y, 0 m	Second cousin
2	Maya	14 y, 6 m	F	9 y, 3 m	Father; mother
3	Eve	11 y, 10 m	F	1 y, 6 m	Brother
4	Finley	14 y, 8 m	M	4 y, 6 m	Sister
5	Sam	14 y, 6 m	F	3 y, 0 m	Mother
6	Anna	16 y, 4 m	F	13 y, 5 m	Aunt
7	Billy	11 y, 11 m	M	10 y, 9 m	Uncle

Note

y = years; m = months; F = female; M = male.

### Procedure

We developed a semi‐structured interview schedule with reference to relevant CD and lifespan research literature, consultation with a young person with CD, and discussions with a clinical psychologist (see supporting information). We piloted this with two young people who met inclusion criteria and consented to take part, to check for question comprehension and to ensure that the questions elicited in‐depth conversations about disclosure of psychological difficulties. We used pilot interviews in the final analysis as we did not make significant changes to the research procedure or interview schedule.

The lead researcher carried out interviews either in person at the participants’ home, or remotely via video‐call software. Interviews were audio‐recorded and lasted between 48 and 85 min. We anonymized and transcribed interviews verbatim. Participants received £5.00 (in cash) as reimbursement for their time.

### Ethics

The Health Research Authority and Health and Care Research Wales approved Research Ethics Committee (REC reference: 19/WA/0323), in addition to the local NHS Trust Research and Development team, which provided research approval. The study was pre‐registered on ClinicalTrials.gov following ethical approval (Identifier: NCT04240340).

Participants were aware of the research prior to consenting and they were given time to consider whether they would like to take part and raise any questions or concerns. We provided a written debrief to participants in a follow‐up email with the contact details of the research team.

### Analysis

The analysis comprised five fluid stages, following guidance from Smith et al. (2009; see Table 2 for further details).

**TABLE 2:** Stages of analysis based on Smith et al. (2009)

Stage	Overview
1	Each interview transcript was repeatedly read and listened to whilst initial, unstructured notes were made denoting powerful recollections of interviews and observations about transcripts
2	Exploratory descriptive, linguistic, and conceptual codes summarizing the experiences described by participants were noted (steps 1 and 2 repeated in an iterative process)
3	Initial notes were reviewed and theme titles that emerged were recorded, considering both specific quotes and overarching narratives for each transcript
4	Emergent themes were clustered within transcripts to identify cluster labels using methods such as subsumption, abstraction, numeration, polarization, and function. Cluster labels were then combined and reduced into master themes
5	Patterns in master themes were identified across cases to identify superordinate and subordinate themes

### Credibility checks

We carried out several validity checks throughout the research process. Secondary researchers individually reviewed a sample of the transcripts to support the primary researcher in reflecting on the development of themes, and to ensure that they could follow theme development from an idiographic to the collective level. We used reflexivity to identify how personal and professional experiences may influence each stage of the study through 1:1 and group discussions (Smith et al., 2009). In addition, the lead researcher employed peer supervision to facilitate a bracketing interview prior to data collection with the aim of eliciting any preconceptions which may influence the nature of questions asked and the interpretation of data analysis (Rolls & Relf, 2006). The lead researcher also kept a reflective journal throughout the development of the research, and following each interview, to keep a record of personal and professional influences and assumptions.

## RESULTS AND DISCUSSION

In line with the flexibility provided by IPA, we decided to merge the results and discussion sections by relating superordinate themes to the extant literature (Smith et al., 2009). This enabled us to consider our own interpretations of the data within both the context of the theme and the wider theoretical and conceptual context.

### Overview of themes

The researchers constructed three superordinate themes from the qualitative data (see Table 3). We will discuss each superordinate theme in turn alongside direct quotations, to consider our own interpretations of the data and how this relates to the wider theoretical and conceptual context (*italics* indicate participant emphasis).

**TABLE 3:** Participant endorsement of superordinate and subordinate themes

Superordinate and subordinate themes	Total	Sarah	Maya	Eve	Finley	Sam	Anna	Billy
1. “As soon as food started going away it was like oh *ok* this is what it is”: Adjusting to the diagnosis within a developmental context								
1.1. “Over time it just‐it feels more normal”: Growing with the condition	6		•	•	•	•	•	•
1.2 "Why can't I just have one?.. Will it really have an effect on me?": The need for adults to scaffold my understanding over time	7	•	•	•	•	•	•	•
1.3 "My friend group is very good…they always like share what they can like sweets and stuff": the importance of others creating (a safe) space for me	7	•	•	•	•	•	•	•
2. "It can either be a huge deal or not a deal at all": How others respond to my condition matters to me								
2.1 "I don't want to make it serious": The power of labels in mitigating stigma	5	•	•	•		•	•	
2.2 "This has kind of been my life": The problem of others co‐creating my identity	5	•	•			•	•	•
2.3 "I didn't want to be different and people to think of me as like this weirdo": Attempts to avoid the anticipated stigma	7	•	•	•	•	•	•	•
3. "I mean I wouldn't just speak to the random guy or girl": Who is my doctor to me?	6	•	•		•	•	•	•

Note

• Indicates that the participant endorsed the theme.

#### “As soon as food started going away it was like oh ok this is what it is”: Adjusting to the diagnosis within a developmental context

This theme encapsulated accounts of participants’ journeys in adjusting to their diagnosis. Participants who were diagnosed in early childhood described an easier intrapersonal integration of CD. For example, one participant was diagnosed at 4 years of age and consequently, CD had shaped his early experiences with food and normalized the absence of gluten from his diet, “It's what I’ve always known because I was so little I don't‐it doesn't really changed how I feel” (Finley). In contrast, those who were diagnosed from early adolescence had incorporated social experiences relating to foods containing gluten (e.g., buying specific treats with their parents at the supermarket) and associated personal and emotional meanings (e.g., memories of eating these foods and the positive emotions that come with this) into their developing self‐concept over several years. Billy received a diagnosis of CD at 10 years of age and describes how this initially symbolized an unwelcome change to his developing sense of self, “If I went shopping I’d see like these coco pop bars that I usually love and I’ll think oh they used to be my *favourite*” (Billy). The use of both present and past tense when describing the self in the context of this moment may reflect a more complex process of intrapersonal adjustment, whereby Billy holds both a narrative of the self “before CD” and “after CD.” Billy describes initial feelings of loss, stemming from the discrepancy between his pre‐diagnosis and post‐diagnosis self. This is perhaps compounded by the context of his developmental stage of identity formation, whereby he is striving to create a continuous sense of identity (Erikson, 1950).

The benefits of early diagnosis were challenged by the need for young people to be provided with developmentally appropriate explanations on an ongoing basis, as they grow with the condition. Participants described a reliance on adults to scaffold their understanding of CD and how to disclose it to others as they age and the dilemmas they faced when this was not provided:

I knew I couldn’t have. I couldn’t have a certain *piece* of food but… I didn’t know what it was so I could say to people that I was gluten free but I couldn’t explain to people…what I *actually* had.(Sam)

Here, Sam's hesitant recall of a time when she first became aware of her diagnosis and her concrete, literal, description of gluten as a “piece of food” indicates the absence of a repeated and updated description of her condition as she has aged. We felt the sense of disconnect that Sam may have experienced when receiving information relating to a defining and unchanging part of her identity “I could say… I was gluten free” without understanding or feeling able to communicate what this really meant about herself or her diet to others. Without this understanding, young people may have difficulty incorporating this diagnosis into their intrapersonal self and presenting this with confidence to the world when asked questions such as, “Why can't you eat that?” or “What can you eat?”.

Without a developmentally appropriate understanding of their condition and the GFD, or language to share this with others, it is unsurprising that young people may struggle with the transition to independently managing their diet as they grow, which can lead to non‐adherence. In attempts to develop their knowledge and confidence in self‐management, some young people described seeking this information via other means, such as the internet. For example, Sarah recalls the moment that she gained an understanding of the possible implications of non‐adherence to the GFD and the implications of this on her self‐management, “I did a whole load of research into it and saw how bad it was and so I was like *I’m not going to do that anymore*” (Sarah). It is possible that adults withhold certain information about the implications of non‐adherence because they do not think the young person would understand or appreciate these risks (Beresford & Sloper, 2003). However, the provision of developmentally appropriate information relating to the importance of the GFD and its implications has been evidenced to increase diet adherence, and thus reduce the risk of future health implications, in young people (Guandalini & Young, 2014).

In line with their growing desire for autonomy, participants described the need for clinicians to adjust their language during appointments so that they could understand and respond to clinicians’ questions. Here, Maya describes a mismatch in the clinicians’ choice of words and her understanding of the information that they are hoping to gather from her:

It’s hard for the questions they do ask me. I *understand*, but I don’t *get* what they are asking me…when it’s passed on to my mum and dad they start the question and they start explaining it and then I’m like, oh I get the question now.(Maya)

This illustrates the importance of clinicians adjusting the language they use when asking questions and scaffolding their information gathering, by embedding questions into examples that are relevant and meaningful for young people in accordance with their stage of development. In the absence of this, participants showed limited understanding of their condition, their diet, or the purpose of medical interventions (such as the antibody blood test) and why this was relevant to them and their health. At 11 years old, Eve described how she had made sense of the blood test in the absence of developmentally appropriate explanations, “[it's] to check how much blood they can get from you without me going pale” (Eve). However, at the age of 16, Anna conveys an awareness of her limited knowledge about the purpose of her medical check‐ups and a feeling of embarrassment associated with this, “there's just blood tests when I go basically…I actually have *no* idea (laughs) like I don't know what they are (laughs)…yeah” (Anna). We interpreted this contrast in their emotional response to being asked about the blood test in the context of their different developmental stages. At 11 years of age, Eve may not feel that others, including the interviewer, expect her to understand and play an active role in the management of her health during check‐ups. However, at 16‐years of age, Anna may be experiencing some psychological dissonance regarding her perception of the interviewer's expectation of her role in managing her condition and her current understanding and ability to do this, resulting in the experience of shame. As young people progress developmentally through paediatric services, they need to be supported by clinicians to acquire appropriate skills, knowledge, and motivation to become well‐informed and committed partners in their healthcare (Dovey‐Pearce et al., 2005). Participants who had experienced this reported feeling better able to explain their diagnosis to others: “They told me…my stomach is like an un‐blown‐up balloon and then when I eat gluten, the balloon blows up…and that's probably the simplest way, I say the exact same thing every time” (Maya). Using the analogy of blowing up a balloon is effective and memorable as it provides a frame of reference that is shared by the young person and her peers.

The importance of having an informed understanding of the condition was reinforced by the need for young people to build up a peer‐based network of “supporters,” who helped them navigate social situations involving food, playing a key role in their acceptance and adjustment to the condition. For many young people “being supportive” meant others’ having an understanding of the GFD so that alternative foods could be provided, and they would not feel left out or forgotten by their peers, “If I’m going to a sleep‐over I’m not just there with like a bowl of milk or something…it makes me feel quite good” (Finley). For others, “being supportive” meant actively taking an interest in their condition and asking about this, “I feel like having that person which you just really talk about it…to have conversations like ‘how's your gluten free’ y'know ‘what's going on’” (Sarah). Here, young people having sufficient understanding of their own condition and diet appears pre‐requisite to cultivating support in others. The absence of this understanding can lead to difficulties correcting others’ misunderstandings and create difficulties in cultivating support. For example, Sam describes how she attempts to convey the seriousness of her condition to others as a justification for her social faux‐pas in declining to try the food that her peers are offering her, but struggles to find the words for this: “They're like “oh can't you try just a little bit just to see what it tastes like” and it's always. It's *no*. because it's not…It's not that simple” (Sam). It is possible that when young people have difficulties finding the language to justify their dietary decisions and cultivate acceptance of this from their peers, it could reinforce the diminished importance that others place on the GFD and reduce the likelihood that others would provide alternatives or ask about the young person's condition “supportively” in the future. Evidence suggests young people who are able to share appropriate knowledge about CD and the GFD with their peers experience reduced stigma around the condition and, in particular, the GFD (Olsson et al., 2009). Hosting group spaces for young people with CD to share information and coping strategies with each other is also associated with better psychosocial adjustment and reduced feelings of alienation and perceived stigmatization (Shani et al., 2020).

#### "It can either be a huge deal or not a deal at all": How others respond to my condition matters to me

This theme also stems from a consideration of developmental context, in that it captures the importance young people place on how others, particularly peers, respond to their condition. When it comes to disclosing the condition, Sarah described hoping to strike a balance between others taking it seriously and providing safe foods, without drawing unwanted attention:

Cus I mean you can be like at two ends of the scale like sometimes when people completely overreact like, “OH MY GOD SOMEONE’S GLUTEN FREE LETS ALL DO THIS BIG THING FOR THEM” and then the other side there’s like I’m in *this situation* and there’s *nothing* gluten free and *no one* cares at all.(Sarah)

Sarah speaks of the perceived social risks she takes when disclosing her dietary needs and the strong emotions she can feel through others’ responses. She paints a vivid picture of others’ either singling her out as different or not seeing or caring about her needs at all. This perhaps reflects her developmental stage, whereby her social self‐consciousness is heightened as she tries to establish a social identity that is also acceptable to others. In striking this balance, participants described facing a conundrum with regards to the label they used to disclose their condition. The label “disease” often drew unwanted attention and could lead to perceived or actual stigma from peers as Sarah goes on to describe, “If I say coeliac disease they're like ‘OHMYGOD you've got a DISEASE’” (Sarah). Similarly, Eve explains, “coeliac disease sounds a lot worse than gluten free…because it's *disease*…It makes it sound as though like, you're gunna *die* from it when really you're not” (Eve). Young people's pre‐conceived connotations and beliefs about words such as ‘disease’ did not fit with their own understanding and experiences of their condition, so many chose to avoid this label to mitigate others’ misunderstanding of their condition and co‐creating their identity in a way that felt problematic. Billy describes his experience of this and how this problematic co‐creation can become internalized over time, “A few people responded in like ‘*oh that's so rubbish’* or ‘that's *terrible*’ then I have in my mind always, you know in the back of my mind that it is gunna be *rubbish*” (Billy). This challenge again speaks to participants’ developmental contexts, whereby young people are both yearning to carve out their personal identity whilst also consolidating their social identity based on their membership in certain social groups (Arnone & Fitzsimons, 2012). This poses the challenge of trying to navigate the complexities of disclosure to avoid taking on an unwanted identity and the perceived or actual stigma resulting from real or imagined differences (Erikson, 1950; Schroeder & Mowen, 2014).

When talking about disclosure to their peers, participants described how they felt others’ may perceive them negatively because their condition made them different. For example, “I just felt like I was just like the odd one out like the *one* friend who couldn't have that specific thing” (Sam), and “obviously in primary school everyone wants to be the same…So it's like are they gunna think I’m *weird*” (Maya). This was sometimes based on experiences of others’ stigmatizing reactions to disclosure in situations involving food, “He still finds it *shocking*. That I can't eat KFC” (Sam) and “they'll be like oh Maya with her gluten free, she can't eat anything” (Maya). CD and the GFD are not always well understood by others, suggesting young people with CD may be more likely to internalize others’ views about their condition being undesirable, abnormal, and something that should be concealed through avoidance (Copelton & Valle, 2009; Schroeder & Mowen, 2014), a coping strategy utilized by some young people in this study, “I didn't tell my friends for ages and I can *remember* that. Like not telling them” (Sarah) and, “on the rare occasions when I have been asked to go out to places to eat I just say no…I’m like very *careful* about it” (Anna).

Alternative coping strategies included reframing the condition as something that others were missing out on, “when I was younger, like when people asked me why I couldn't eat things I’d say because I’m *special*” (Eve) and, “I sort of say quite a lot of gluten free food is *better* than food with gluten in it” (Billy). By reframing the condition and the GFD as a strength or assert, it reduces the likelihood that others will perceive these as weaknesses or as undesirable (Kaushansky et al., 2017). Alternative strategies used to incorporate CD into their identity in a stigma‐reducing way included educating their friends and family about the condition to ‘bring others onto their side’ and curate self‐acceptance. This approach was successfully used by Maya, who felt skilled in explaining her condition and diet to others, “They're like oh that's fine and, accepting that I can't eat it and, it's not personal” (Maya). Sam describes the need to first hold others’ minds in her own to make sense of how they might perceive the condition and diet and then, from this, scaffold their understanding of the condition to align with her own:

They may have heard the term gluten‐free but I don’t think they’ve ever like *asked* like what it *actually* was. So they just never really *learn*…they’re just like “oh *what’s* that?” and then they *understand* and then they take it seriously.(Sam)

Sam's ability to mentalize with her peers in this way enables her to respond with empathy and openness when confronted with misinformed, or potentially stigmatizing beliefs about her condition. This strategy is similar to the “neutralizing motive” set out by Sharp (2009), whereby disclosure of and education about a condition was found to minimize the effects of stigma. This approach has been evidenced to help young people maintain a positive social identity and can result in greater understanding and solidarity in their social groups (Schroeder & Mowen, 2014).

#### "I mean I wouldn't just speak to the random guy or girl": Who is my doctor to me?

The researchers felt that this theme encapsulated the barriers that young people identified to disclosing psychological difficulties to clinicians. Some young people held stereotypical beliefs about doctors as serious and strict, which deterred young people from sharing their feelings with them. For example, Sam explains, “They may think that if they tell them like they may be angry about it because they don't have that like gentle face, they have a very serious face” (Sam). This demonstrates the importance of non‐verbal communication in either reducing or enhancing the power dynamic between young people and their doctors and what this consequently means to young people about perceived safety in disclosure. Similarly, Maya describes ascribing doctors with high status and positioning them as “knowing everything.” This contrasts with a sense of her own inferiority, which generated feelings of anxiety:

I definitely myself get nervous…if I’m talking to doctors…it’s very intimidating when someone’s sitting there and asking you is *this* wrong with you…you’re sitting there talking to an adult who knows everything, and you don’t know anything yourself. That’s the scariest part of it.(Maya)

Here, Maya suggests that her view of doctors as knowledgeable authority figures can exacerbate her experience of the power dynamic in the consultation room. Through assigning the doctor with this status, Maya paints a picture of experiencing the consultation as a test, whereby she is anxious about both getting the answers wrong and her perception that there could be negative consequences of this, for example, being negatively judged by the doctor. This suggests that doctors need to play an active role in redressing the power balance early in the consultation, before questions are asked, so that the young person feels able to fully engage in discussion about their condition without anxiety about getting things wrong (Ludvigsson et al., 2016).

Many young people, such as Anna, held beliefs about what doctors are and are not interested in hearing about and this often led them to anticipate that medical consultations would not be an appropriate place to disclose emotional difficulties:

These people are here to help me for medical reasons… if someone was struggling I don’t think they’d even *think* about a doctor like being an outlet to explain their emotional like problems with it.(Anna)

Anna describes young people as seeing the biological impact and management of CD as separate from the psychosocial and therefore reasons that many young people would assume that different clinicians should be approached to meet these separate needs. When considering the wider context of health services this is unsurprising as, despite strong evidence that the biopsychosocial model can lead to more effective outcomes in chronic conditions, it is largely under‐implemented in such services because of time‐ and resource‐constraints in addition to limited clinician experience in formulating in this way (Hatala, 2012). Therefore, young people's experiences of medical consultations may not always be integrated, which will shape their understanding of what is and is not appropriate to disclose to medical doctors.

There is evidence that young people who have established and trusted relationships with clinicians are more likely to seek help in the future (Gulliver et al., 2010; Rickwood et al., 2007). Thus, there is a need for clinicians to actively break down barriers. For example, Billy describes how doctors do this effectively by adapting their body language to communicate safety and time:

They don’t just ask you a question and then do something else but they’ll pay attention to it…they usually like turn around on their chair and push out from their computer and that just shows that they’re not gunna be doing anything on the computer.(Billy)

Billy notices cues from the clinician that reduce the power dynamic and enable him to establish trust in knowing that he can share his experiences and that these will be listened to.

Young people were often aware of the stress on the wider hospital system and either felt that the way they were feeling was not important enough to take up the clinician's time or that they would be seen by the clinician as a timewaster, “I didn't want people to think I’m – not weak, but I didn't want people to think I was like *struggling* with something which is supposedly y'know outlook and seen as so just *easy*” (Sarah) and, “They're like doctors and they just wanna like‐ they've got a lot of people to see they just wanna do the session and move on” (Anna). Here, we can see young people placing greater importance on these factors than on their own needs. Children are often expected to, and rewarded for, adjusting their behaviour to meet their perception of adults’ needs and expectations and can fear being negatively judged by them if they do not do this (Ludvigsson et al., 2016). During consultations, it is often parents who share more difficult symptoms or assert the young person's needs (Vatne et al., 2012). As young people transition to adulthood, there is a need for them to also develop their own confidence and ability to assert and seek relevant support for their needs (Ludvigsson et al., 2016).

In line with existing research, few participants recalled being explicitly asked about their emotional adjustment during consultations and suggested that it would be helpful for clinicians to provide clear permission to disclose psychological difficulties, so that they could recognize and respond to them accordingly (Crocker et al., 2020; Radez et al., 2020):

I feel like when the doctors are *speaking* maybe there should be like an extra question like “how are you feeling?”…every time I think they say “how are you going with it?” I always assume they’re asking me like how am I finding alternatives…I don’t actually think they’re asking me like *emotionally* like “how do you feel?”.(Anna)

Anna's perception of doctors’ state of mind may be informed by her own understanding and experience of what doctors are interested in hearing about. Attending a medical appointment about her physical health condition understandably primes her expectation that she will be asked questions about her physical symptoms and practical management of the diet. Additional behavioural and contextual cues are required to demonstrate an interest in psychosocial factors to broaden this understanding. In the absence of this, young people may continue to hold the assumption that they should not be experiencing difficult feelings, clinicians are not interested in this, or that their appointment is not the place to disclose this, as described by Sarah:

[the doctor] has always just sort of been like so “how’s your stomach feeling?”…it’s never been like “how are *you* feeling?” so like I didn’t feel like it was the right *place* for me to talk about my feelings to them. Um or that I *should* be feeling those feelings about something so (pause) *small*.(Sarah)

Sarah describes feeling unable to disclose her feelings to her doctors. She draws on her experience of condition‐centred consultations in her construction of the “status quo” and uses this to guide her behaviour so as not to disrupt this in a situation where she may already feel inhibited by an unequal power dynamic. Sarah feels a lack of validation for her feelings, having not felt able to share them, which may lead her to draw self‐stigmatizing conclusions about the validity of her own experiences and whether she “should” be feeling that way.

### Summary of findings

Participant narratives of adjusting to a diagnosis of CD and the GFD were influenced by their stage of identity formation at the point of diagnosis, together with their access to developmentally appropriate information about the condition and the GFD. Erikson (1950) proposed that “identify formation” occurs during adolescence, whereby young people strive to create a continuous sense of identity through experimenting with different activities, roles, and behaviours in a context whereby they are becoming more autonomous in the decisions they make, including their diet and health management. Participants who experienced symptom onset and diagnosis of CD during this developmental stage experienced “biographical disruption” in how they saw themselves which often led to the development of a pre‐/post‐diagnosis dichotomy (Bury, 1982). In line with other CD studies, this experience was compounded by their social context, as they were required to grapple with dietary changes alongside increased independence in eating out with friends and reduced supervision with greater choice over lunches at school (White et al., 2016). In contrast, young people diagnosed earlier in childhood are at an earlier developmental stage of identity diffusion, whereby they had not yet committed to a particular identity, in a context where adults often take responsibility for creating environments in which safe foods are provided (Marcia, 1980). In this social and developmental context, these young people described being better able to integrate the diagnosis into their developing identities to create a more cohesive narrative.

In accordance with the common‐sense model (CSM) of illness perceptions, the absence of developmentally appropriate information about their condition meant that young people had to rely on lay representations of what their condition meant to guide their disclosure and understanding (Leventhal et al., 2003). Stronger “illness coherence” and a sense of “personal control” over the condition and the diet have previously been linked to improved health‐related quality of life and self‐management in adults with CD (Ford et al., 2012). As CD is not widely known or understood in the same way as other chronic conditions, such as diabetes, young people may not have an accurate pre‐existing or “lay” representation or understanding of the condition to develop a sense of coherence and control (White et al., 2016), emphasizing the important role of adults in providing this developmentally appropriate information to help shape these representations and corresponding health‐related behaviours. Recent evidence suggests that a good knowledge of CD is positively related to GFD adherence and positive quality of life (Zingone et al., 2018).

Participants who described difficulties explaining and normalizing their condition, or who had a motivation to conceal their condition, experienced a sense of feeling discriminated against when it was made visible through situations involving food, through others either minimizing or over‐emphasizing the importance of their dietary needs. Disclosing the GFD as an adolescent can pose a threat to efforts to conform to their social group by identifying differences (Arnone & Fitzsimons, 2012). Lay knowledge about dietary requirements is improving in society, however, knowledge about CD and the importance of a strict GFD remains sparse (King et al., 2019). Modified labelling theory suggests that when diagnosed with an incurable condition that creates a difference in a context of limited understanding from others, it can lead to internalized experiences of stigma (Copelton & Valle, 2009; Schroeder & Mowen, 2014). However, having a good understanding of their condition and support from peers in finding foods they could eat in social situations counteracted the young person's perception of being “different,” in line with previous literature exploring the experiences of CD in young people (Olsson et al., 2009; White et al., 2016). Furthermore, in accordance with the CSM, holding positive representations of CD can have a direct impact on an individual's coping, positively impacting emotional well‐being and health outcomes (Leventhal et al., 2003).

This study adds to the existing literature by considering barriers to the disclosure of psychological difficulties associated with CD in adolescents. Young people's ability to communicate sensitive concerns relating to their condition to clinicians is key in their transition to self‐management and independent attendance at medical appointments (Ludvigsson et al., 2016). Participants described experiencing a negative power dynamic in the presence of doctors and experienced consultations as condition‐focused. In line with existing research carried out with young people with other chronic conditions, the young people in this study suggested that this power dynamic was maintained by doctors using inaccessible language and focusing on dietary adherence or physical symptoms associated with non‐adherence, as opposed to other psychosocial factors that may be relevant to condition management (Beresford & Sloper, 2003). In accordance with a literature review exploring adolescent experiences of healthcare professionals, the communication style of the doctor was also cited by participants as important in reducing the power‐dynamic and building trust through being personable and interested (Davison et al., 2021). Here, young people also described making judgements about the approachability, availability, and interest of the doctor from their non‐verbal communication skills and environmental cues present in the consultation room (Hawthorne et al., 2011). Existing literature suggests that young people hold beliefs that they should be able to manage their own emotional difficulties, and may perceive help‐seeking as a sign of weakness (Radez et al., 2020; Rickwood et al., 2007). Thus, permission‐giving is equally important when young people are doing well with adhering to the diet, to normalize that they may still experience difficult emotions relating to the condition and that this is not a reflection of them not managing well. All identified barriers to disclosure were in line with previous research, suggesting that they are not specific to young people with CD and the following recommendations could have a wide‐reaching effect across paediatric services (Beresford & Sloper, 2003; Gulliver et al., 2010; Radez et al., 2020).

### Recommendations for services

We generated theoretically driven recommendations based on findings to support young people's understanding of CD and the GFD, provide them with the language to disclose and explain this to others, and break down perceived barriers to the disclosure of psychological difficulties to clinicians. We gathered feedback on these recommendations through discussion with members of the medical team in the present service and incorporated this feedback into the discussion.

#### Recommendations regarding the provision of developmentally appropriate information

Young people described the need for information to be updated and repeated at each appointment in accordance with their developmental stage, to enable them to develop “illness coherence” to support the integration of the condition into their identity and enable them to disclose the condition in ways that would minimize their experience of stigma. Findings suggest that a proactive and sensitive approach to providing updated information during appointments is required as young people can feel embarrassed or lack confidence in asking for this information themselves. Providing information in a leaflet that goes out to patients ahead of appointments explaining what the appointment and blood test are for in accordance with their developmental stage may enable this information to be shared. This method acknowledges the time constraints of routine consultations, which may make it difficult for clinicians to provide this updated information themselves. Notwithstanding this, findings suggest that during the appointment it may also be helpful for clinicians to normalize forgetting and provide key information again, for example by stating, “Often people forget what the blood test results mean, so here is the information again.”

Young people described how the lack of an age‐appropriate understanding of their condition could lead to avoidance of disclosure to peers and adults and, occasionally, non‐adherence to the GFD. Recent research in Italy has developed the “TRANSIT‐CeD disk, a tool for clinicians to measure a young person's CD and GFD knowledge, ability to self‐manage their diet, diet adherence, and QoL and identify areas for further development” (Zingone et al., 2018). Future research could explore whether the use of this tool in UK samples may mitigate these risks as they transition to self‐management of their condition.

An additional way of sharing this information could be through the service facilitating education days, to equip young people with the knowledge and language to share this information with their peers. In line with findings, education days could take place at different developmental stages such as during years 5 or 6, ahead of transitioning to secondary school, and again in years 9 or 10, as young people develop greater autonomy over their diet. As an alternative to this, one young person suggested developing an educational video, or resources for teaching about the condition in schools as a way of making it easier for young people to talk about how the condition makes them feel. This may also support young people in developing a network of “supporters” to help them navigate social situations involving food and minimize feelings of stigma. Future research could explore the impact of these interventions on perceived stigma in this population.

#### Recommendations regarding how to actively break down perceived barriers to disclosure

Young people reported an assumption that if they were not asked about their emotional well‐being, then this must be because it should not be spoken about, or it was something that doctors were not interested in hearing about, in line with their experience of condition‐focused consultations. Sending written information to young people in advance of their appointment to explain that a hospital is a place where they can go to talk about how they feel, to bring this into the young person's awareness before they arrive at a hospital for their appointment may be helpful. Several young people also suggested that it would be helpful for clinicians to provide clear permission to disclose psychological difficulties during appointments, by asking an extra question about how young people are feeling emotionally. This is in line with evidence reinforcing the need for disclosure to be scaffolded by others (Crocker et al., 2020; Radez et al., 2020). For example, clinicians could ask, “Sometimes young people tell me that they worry about eating out with their friends, is this something you worry about sometimes too?” This could be followed up by the use of a screening tool if mental health difficulties are indicated.

When this feedback was discussed in the current service, clinicians shared concerns that opening‐up conversations about emotions would lead to requests for referrals to psychology at a rate that could not be managed by existing psychological provision and that they therefore felt they should be prioritizing access to psychology for young people with conditions such as IBD, whereby physical symptoms are more unpredictable in nature and may require more invasive medical intervention such as surgery. This reflects very real limitations in service provision but may also validate participants’ experiences of feeling that their condition is perhaps, “Something which is supposedly y'know outlook and seen as so just *easy*” (Sarah) in comparison to other conditions. Through discussion, clinicians were made aware that most young people would likely not need psychological input but would benefit from having their feelings validated and normalized and perhaps being directed towards self‐help resources. This would be in line with a stepped care design, which would consider the most efficacious use of resources in a busy paediatric hospital setting (Hamall et al., 2014; NICE, 2019).

Although some young people described experiencing a supporting and containing power dynamic during appointments by positioning the doctor as an “expert,” many young people disclosed experiencing a negative power dynamic in their appointments, describing themselves as feeling inferior, or at risk of being “told off.” Many also had pre‐existing assumptions about the role of a medical doctor which did not include talking about emotions or coping. Supporting the MDT to explicitly ask about psychosocial well‐being in consultations to clearly show the young person that they have space and time for discussion, understanding, and empathy of emotions could help redress unhelpful power dynamics and break down pre‐existing stereotypes. One way of doing this may be to put an “emotion poster” on display in the consultation room. The clinician could then use this to ask the young person which picture represents the way they are feeling about their condition and share the emotion that they are feeling that day too. Young people also commonly described the importance of the clinician's body language in developing their perception of how “safe” and interested they were. Specifically, young people were often keen to please clinicians and were attuned to their facial expressions, whether the clinician asked questions whilst typing notes on the computer or whilst facing the young person with their full attention, and whether the clinician was addressing questions to the young person themselves, or their parents/guardians. Developing and collaboratively delivering specific, experiential training with young people for doctors on the importance of non‐verbal communication skills, such as eye contact, facial expression, tone of voice, and posture on building rapport and demonstrating warmth and safety may increase their understanding and use of this.

## CONCLUSIONS

This is the first UK study to directly explore the barriers perceived by young people with CD to disclosing psychological difficulties to clinicians during routine consultations. Findings highlight the importance of clinicians providing developmentally appropriate information to young people and actively breaking down barriers through body language and the use of clear questions regarding emotional experiences. Future research could explore the impact of implementing the stated recommendations in services on young people's understanding of their condition, overall patient experience of routine consultations, and the identification of patients who may benefit from psychological support in adjusting to their condition.

Findings demonstrate an alignment in experience between the seven participants interviewed, with quotes drawn reasonably equally from participants. This suggests that we recruited a homogenous sample, whilst applying commitment and rigour in our approach to analysis (Yardley, 2000). On the one hand, recruiting a sample comprising white British participants receiving support from a single service enabled us to ensure sensitivity to participants’ developmental and sociocultural context (Yardley, 2000). However, this may limit the impact and application of findings across cultures and services and future research should give voice to additional underrepresented samples. Several validation methods and credibility checks were used, however, the analysis presented is the researchers’ interpretation of the data and other interpretations are possible.

This study emphasizes the value of in‐depth, qualitative feedback from patients in identifying areas for service improvement. Although the provided recommendations are made based on young people's experiences of the present service, evidence suggests that the barriers identified are not specific to young people with CD. Therefore, it is hoped that findings may positively benefit patient experience and improve access to psychological support in both other paediatric gastroenterology services and other healthcare settings.

## CONFLICTS OF INTEREST

No potential conflict of interest was reported by the authors.

## AUTHOR CONTRIBUTIONS

**Juliet Kennedy** (Methodology; Resources; Supervision; Validation; Writing – review & editing) **Matthew Knight** (Conceptualization; Methodology; Supervision; Validation; Writing – review & editing) **Miranda Wheeler** (Conceptualization; Data curation; Formal analysis; Investigation; Methodology; Project administration; Validation; Visualization; Writing – original draft; Writing – review & editing) **Annabel L. David** (Conceptualization; Methodology; Resources; Supervision; Writing – review & editing).

## Supporting information

Supplementary Material

## Data Availability

Raw data, beyond those displayed in this article, are not shared.
