# Supplementary material for: “I sort of never felt like I should be worried about it or that I could be worried about it’” an interpretative phenomenological analysis of perceived barriers to disclosure by young people with coeliac disease
Source: Br J Health Psychol. 2022 May 16;27(4):1296–313. doi: 10.1111/bjhp.12599 (PMC9790695; doi:10.1111/bjhp.12599)
Supplement: Supplementary file 1 — Supplementary Material [file BJHP-27-1296-s001.docx]

**Supplementary Information A**

**Semi-Structured Interview Schedule**

**SIP Interview Schedule:**

**Introduction questions/ experiences of being a young person with coeliac disease**

1. To start with, I would like to find out a bit about you. Tell me about your hobbies or interests?

**Prompts:** Are you into sport/ music etc.?

1. Can you tell me a bit about what it’s like to have [coeliac disease]?

**Prompts:** Do you think [coeliac disease] has affected your relationships with your family or friends? How? How has it affected your day to day activities or hobbies? Has it caused any problems? What happened? How did you feel? How did you manage?

1. Can you tell me when you first became aware that you had [coeliac disease]?

**Prompts*:** What happened? What kind of physical problems did you notice? How did that feel in your body? What did you think was happening?

********Here the interview can progress down one of two pathways: 1. If they talk about medical appointments/diagnosis then can move onto ‘Experiences with Children’s services’ after prompts; 2. If they don’t continue with current section.*

1. What impact has having [coeliac disease] had on how you are feeling emotionally?

**Prompts**: How has it made you feel about yourself? Has it ever had a negative effect on how you are feeling emotionally? Has it ever had a positive effect on how you are feeling emotionally?

******* *Here the interview can progress down one of two pathways: 1. If they say it has negatively affected their wellbeing then can skip to ‘What is your experience of talking to your doctor about how you feel about coeliac disease?’; 2. If they say it has not negatively affected their wellbeing then continue in this section.*

1. How have your thoughts and feelings about [coeliac disease] changed over time?

**Prompts:** How has your experience of having [coeliac disease] changed over time, as you are getting older? How important is [coeliac disease] to you? How important does it feel for you?

1. Who have you told about your [coeliac disease]?

**Prompts*:** Who did you first tell about your diagnosis? How do you tell them? Who did you speak to? How did they respond? What has been helpful about telling others? What has been unhelpful about telling others? Do you think telling others has changed how they see you? Has telling these people changed things for you? Are there people you can’t tell or don’t want to tell? Why?

********Here the interview can progress down one of two pathways: 1. If they talk about medical professionals, can move onto ‘Experiences with Children’s services’; 2. If they talk about friends/family/school staff can continue in this section.*

1. What do you say when you talk to others about your [coeliac disease]?

**Prompts:** Do you talk to anyone about how it makes you feel? Are there things that you feel worried about talking to others about relating to your [coeliac disease]? How do you feel about telling people about the problems you go through? Does that change? Has that changed over time? Are there things you don’t want to talk about with other people?

1. How do you think people with [coeliac disease] are seen by others?

**Prompts:** How is [coeliac disease] seen by your parents/ family/ teachers/ friends/ healthcare professionals? Do people treat you differently to others? How seriously is [coeliac disease] taken by others? How do you think [coeliac disease] is viewed compared to other health conditions, like a asthma or other allergies (by family, friends, school (peers and teachers) and healthcare professionals)?

**Experiences with Children’s services**

I’d now like to ask you about your experiences of the gastroenterology service, at the Oxford Children’s Hospital, can you remember which service I’m talking about?

1. What is/was it like to go to appointments at the paediatric gastroenterology service at the children’s hospital?

**Prompts:** When appointments come up how does that make you feel emotionally? How does/did it feel being there? What does it feel like afterwards? What do you think the appointments are for? Is there anything you like about the service/ think that they do well? Is there anything you don’t like about the service/ think that they could do better?

1. What do you think your doctors in the gastroenterology service at the Children’s Hospital are interested in hearing about?

**Prompts:** What is it that makes you think that? What is that like for you?

1. What is your experience of talking to your doctor about how you feel about [coeliac disease] or talking to them about [insert shared difficulties]?

**Prompts:** How did you let them know how you were feeling? What made this easier? What made this more difficult? What might make you feel unable to talk to your doctor about this? Do you feel more able to talk to certain professionals than others?

1. Thinking about other young people with [coeliac disease], what do you think might make them feel unable to talk about their difficulties (for example, with following the gluten free diet, or with concerns about social situations that involve food, or feeling different to others or feeling worried) to doctors in the service?

**Prompts:** Do you think your experience of talking to doctors in the service is the same as others?

**Service improvements**

1. What could the service do to make it easier to talk about how you feel?

**Prompt**: How do you think that might help?

**Ending**

1. Is there anything that we haven’t talked about that you would like to mention about your experience of having [coeliac disease]?
2. Anything that I haven’t asked about that you think is important for me to know related to your experience of the service?

Thank you for taking the time to talk to me today and for talking about your experiences so openly.

[turn recorder off]

[Give participant and parent (where applicable) debrief and allow time for any questions they may have about the study or their participation].

[Reimburse the parent for any travel costs and the participant £5 for their time, ensure signed receipt for payment is taken].

**Supplementary Information B**

**Reflexivity and Positioning of all Authors**

**Situating the Researcher: XX**

**Position on the Research Area (Personal and Professional)**

XX is a White British Female in her 20’s without any gastrointestinal or autoimmune conditions. Her primary clinical and research interests include working with people with chronic health conditions and she has several years of experience researching and supporting both adults and children in the context of adjusting to long term health conditions. With regards to personal experiences, a member of her family has type 1 diabetes and coeliac disease, both of which were diagnosed during adolescence. XX has no prior experience of conducting research using IPA.

**Situating the Researcher: XX**

**Position on the Research Area (Personal and Professional)**

XX is a Clinical Psychologist in a Paediatric Gastroenterology Service. She works with children and adolescents with Coeliac Disease and their families. Her research interests are the impact of living with chronic health conditions, especially lived experiences of gastrointestinal diseases, stigma, and psychosocial wellbeing. XX is a white British female in her 30s who does not have Coeliac Disease.

**Situating the Researcher: XX**

**Position on the Research Area (Personal and Professional)**

XX is a White British female in their 30’s, with no lived experience of Coeliac Disease. XX is a Clinical Psychologist working clinically over the past 8 years in a paediatric medical setting. XX has an interest in using IPA research within the paediatric health setting.

**Situating the Researcher: XX**

**Position on the Research Area (Personal and Professional)**

XX is a White British male in their 40’s, with no lived experience of Coeliac Disease. XX is a Clinical Psychologist with academic, clinical and research interests in enduring health conditions and potential public-stigma, self-stigma, and ramifications thereof. XX has experience of conducting IPA with Experts by Experience, Experts by Profession, and carers/relatives.
